# Simulating the restoration of normal gene expression from different thyroid cancer stages using deep learning

**DOI:** 10.1186/s12885-022-09704-z

**Published:** 2022-06-04

**Authors:** Nicole M. Nelligan, M. Reed Bender, F. Alex Feltus

**Affiliations:** 1 Department of Genetics & Biochemistry, Clemson University, Biosystems Research Complex, 302C, 19 105 Collings St,., SC 29634 Clemson, USA; 2 Biomedical Data Science and Informatics Program, Clemson, SC 29634 USA; 3 Clemson Center for Human Genetics, Greenwood, SC 29646 USA

**Keywords:** Thyroid cancer, Deep learning, Transcriptome, TSPG

## Abstract

**Background:**

Thyroid cancer (THCA) is the most common endocrine malignancy and incidence is increasing. There is an urgent need to better understand the molecular differences between THCA tumors at different pathologic stages so appropriate diagnostic, prognostic, and treatment strategies can be applied. Transcriptome State Perturbation Generator (TSPG) is a tool created to identify the changes in gene expression necessary to transform the transcriptional state of a source sample to mimic that of a target.

**Methods:**

We used TSPG to perturb the bulk RNA expression data from various THCA tumor samples at progressive stages towards the transcriptional pattern of normal thyroid tissue. The perturbations produced were analyzed to determine if there are consistently up- or down-regulated genes or functions in certain stages of tumors.

**Results:**

Some genes of particular interest were investigated further in previous research. *SLC6A15* was found to be down-regulated in all stage 1–3 samples. This gene has previously been identified as a tumor suppressor. The up-regulation of *PLA2G12B* in all samples was notable because the protein encoded by this gene belongs to the PLA2 superfamily, which is involved in metabolism, a major function of the thyroid gland. *REN* was up-regulated in all stage 3 and 4 samples. The enzyme renin encoded by this gene, has a role in the renin-angiotensin system; this system regulates angiogenesis and may have a role in cancer development and progression. This is supported by the consistent up-regulation of *REN* only in later stage tumor samples. Functional enrichment analysis showed that olfactory receptor activities and similar terms were enriched for the up-regulated genes which supports previous research concluding that abundance and stimulation of olfactory receptors is linked to cancer.

**Conclusions:**

TSPG can be a useful tool in exploring large gene expression datasets and extracting the meaningful differences between distinct classes of data. We identified genes that were characteristically perturbed in certain sample types, including only late-stage THCA tumors. Additionally, we provided evidence for potential transcriptional signatures of each stage of thyroid cancer. These are potentially relevant targets for future investigation into THCA tumorigenesis.

**Supplementary Information:**

The online version contains supplementary material available at 10.1186/s12885-022-09704-z.

## Background

Thyroid cancer is the most common endocrine malignancy, with an estimated 44,280 new cases resulting in over 2,000 deaths in 2021 [1, 2]. The incidence of thyroid cancer is increasing worldwide with an annual percent change around 6% in recent years [3]. This increase is at least partly due to increasing diagnostic capabilities through novel imaging techniques that can easily be used to detect potentially malignant thyroid nodules [4]. Early detection of thyroid cancer helps to reduce overall mortality, but over-diagnosis may cause individuals who would not have developed malignant cancer to undergo unnecessary treatments, subjecting them to procedural risks and financial burdens [5]. Thyroid cancer, however, can present in aggressive variants that grow rapidly, metastasize, and negatively impact normal functions like breathing and swallowing if left untreated [4]. Therefore, it is important to differentiate late-stage and more aggressive THCA tumors so patients can receive the appropriate treatment.

A common treatment for thyroid cancer is thyroidectomy: partial or complete removal of the thyroid gland [5]. The thyroid normally produces thyroid hormone which is essential for proper growth and regulation of metabolism [6]. Considering its importance for maintaining normal functions, the unnecessary loss of this gland should be avoided to optimize patients’ quality of life. Later stages of well-differentiated thyroid carcinomas, especially stage 4, are associated with higher risk of recurrence and more aggressive variants, making this a necessary treatment despite the ultimate burden [7].

Well-differentiated thyroid cancer, with papillary thyroid cancer as the most prevalent form, most often involves genetic alterations that constitutively activate the mitogen-activated protein kinase (MAPK) cascade, especially chromosomal rearrangement of *RET* and point mutation of *BRAF* or *RAS* [7, 8]. Thyroid cancer may also be triggered by an overactive phosphatidylinositol-3 kinase (PI_3_K/AKT) pathway due to activating mutations in *RAS*, *PIK*_*3*_*CA*, or *AKT*_*1*_, or inactivation of *PTEN* [9].

There is an urgent need to better understand the molecular differences between THCA tumors at different pathologic stages. Past research in breast cancer revealed evidence of distinct gene expression levels in different tumor grades [10]. Previous studies have predicted cancer stage using machine learning techniques with clinical and pathological datasets [11, 12]. In this study, we used a novel deep learning tool, which has been successfully applied to a previous cancer study, to observe abnormal RNA expression levels in individuals with various stages of THCA and find potential signatures for each stage [13]. Linking these signatures to accurate diagnostic, prognostic, and treatment strategies is of high importance.

The Transcriptome State Perturbation Generator (TSPG) is a tool created to leverage generative deep learning for the detection of changes in gene expression needed to transform a labeled *source* sample into the feature space of another, *target* sample type [13]. Using RNA-Seq feature data from labeled sample groups, TSPG first trains a deep learning model to classify samples based on their true class label. In this case, the model is trained to make a prediction about the pertinent label for a given transcriptomic expression vector. Given an unlabeled RNA-Seq vector, this model would learn to predict the true label. Then, an adversarial neural network is trained to subtly perturb those expression vectors so that the classification model will make an errant prediction, instead classifying the perturbed sample as an assigned target class. It does this by changing the transcriptomic profile of the provided sample to look like that of the target class. By examining the most significantly perturbed genes, one can identify differently transitioned genes. Since the deep learning model relies on feature integration at multiple layers, the gene expression patterns of an input gene list (e.g. all genes) are tweaked across the whole distribution. Reducing the gene set is certainly possible by using a limited input gene list for training. For example, TSPG has previously been tested on the Hallmark gene list subset from the Molecular Signatures Database (MSigDB) [13].

We demonstrate that TSPG can learn how to change the gene expression patterns of individual THCA tumors to reflect the expression profile of normal thyroid tissue. This method has previously been used to identify transcriptional aberrations for a specific patient diagnosed with papillary renal cell carcinoma [13]. We are interested in the precision medicine applications of this tool for thyroid cancer, so we have applied this technique to THCA tumors of different stages. While the expression levels are considered and perturbed separately for each individual tumor, the results were combined to identify patterns among stages of THCA tumors. We considered RNA expression data in this study while much of the existing similar research on THCA utilized medical imaging data or clinical attributes [14, 15]. Previous studies have used machine learning methods with transcriptome sequencing data in different cancer types to investigate things like tissue of origin and staging, but this study uniquely reports such results for thyroid cancer [16, 17].

In this study, we used TSPG to perturb the RNA expression data from individual thyroid tumor samples at various stages towards normal thyroid tissue gene expression. The perturbations produced were analyzed to determine if there are consistently up-regulated or down-regulated genes in the various stage progressions of THCA tumors. We have thus provided evidence for potential transcriptional signatures of each stage of thyroid cancer.

## Methods

*Preparation of TSPG input data*. Normalized and batch corrected FPKM gene expression matrices (GEMs) were downloaded from an existing dataset for The Cancer Genome Atlas (TCGA) THCA tumor samples, TCGA normal thyroid tissue, and Genotype-Tissue Expression (GTEx) normal thyroid tissue [18]. One GEM was formed by merging the three GEMs and then it was log2 transformed and quantile normalized using GEMprep [19]. This GEM contained RNA-Seq expression levels for 19,239 genes in 51 TCGA solid tissue normal thyroid samples, 318 GTEx solid tissue normal thyroid samples, 244 TCGA stage 1 THCA solid tissue tumor samples, 47 TCGA stage 2 THCA solid tissue tumor samples, 95 TCGA stage 3 solid tissue tumor samples, and 46 TCGA stage 4 solid tissue tumor samples. Biospecimen and clinical data were downloaded from GDC data portal for all TCGA THCA samples (Table S1 and Table S2). The American Joint Committee on Cancer (AJCC) pathologic stage was matched to the corresponding subject identifier using VLOOKUP in Excel.

*Perturbation of samples toward GTEx normal thyroid*. Transcriptome State Perturbation Generator (TSPG) was utilized to perturb the RNA expression values of THCA tumor samples toward the RNA expression values of normal thyroid samples [20]. Ten of each sample type (stage 1 tumor, stage 2 tumor, stage 3 tumor, stage 4 tumor, TCGA normal thyroid, and GTEx normal thyroid) were randomly selected and removed from the training dataset. The list of samples with their pathologic stage and primary diagnosis is available in Table S3. Labels files were produced in the correct format to label each sample ID as tumor-s1, tumor-s2, tumor-s3, tumor-s4, normal-tcga, or normal-gtex based on the clinical data described above. The GEMs were formatted as required by converting to numpy arrays and transposing using GEMprep. GTEx normal thyroid tissue was used as the target class.

*Analysis of sample perturbations made by TSPG*. From the perturbations file produced by TSPG, significantly perturbed genes in each sample were defined as those having a perturbation value greater than 2 standard deviations above or below the mean of all perturbation values for that individual. The appropriate genes were extracted for each of the sixty perturbed individuals and saved in text files. Then, the consistently tumor-upregulated or tumor-downregulated genes in each sample type were determined by identifying genes that were significantly negatively or positively perturbed, respectively, in all 10 samples from that class. The average number of tumor-upregulated (negatively perturbed) and tumor-downregulated (positively perturbed) genes were calculated for each class using the corresponding ten perturbed samples. Additional calculations included the average number of tumor-upregulated and tumor-downregulated genes shared between two samples of the same type and, in each direction, the average proportion of perturbed genes shared to the total number of unique perturbed genes between two samples of the same type. Bar charts visualizing each of these were produced in Excel.

*Functional Enrichment*. Functional enrichment was performed using FUNC-E [21]. Tumor-upregulated and tumor-downregulated genes from each sample type were used as different modules. Query lists contained the significantly positively perturbed and significantly negatively perturbed genes, separately, in any sample from each sample type. The genomic background was all genes contained in the GEM. A terms list with Gene Ontology (GO), Interpro (IPR), and Kyoto Encyclopedia of Genes and Genomes (KEGG) vocabularies was generated [22–24]. The term mapping list was generated by downloading tab-separated value (TSV) files from ensemble biomart containing Gene name and one of GO term accession, KEGG Pathway and Enzyme ID, or Interpro ID. These TSVs were then merged into one file and filtered to contain only genes present in the genomic background and only terms present in the terms list. The p-value cutoff for enrichment used was 0.01, but the resulting enriched terms were filtered for a Bonferroni corrected p-value of less than 0.00001.

## Results

The classes included in this paper are stage 1 THCA tumor, stage 2 THCA tumor, stage 3 THCA tumor, stage 4 THCA tumor, normal thyroid tissue originating from The Cancer Genome Atlas (TCGA), and normal thyroid tissue originating from the Genotype-Tissue Expression (GTEx) repository. We were able to unify the TCGA and GTEx samples into a unified matrix thanks to the work done by Wang et al. [18]. They have supplied a database of batch-corrected and re-normalized samples from both datasets, so the normal samples from TCGA and those from GTEx could potentially be studied as a single data source. However, we maintained TCGA normal and GTEx normal thyroid tissue as two separate groups in this analysis based on preliminary results suggesting some difference between them. An initial t-SNE plot created using the unified matrix showed TCGA normal samples forming a separate cluster from the GTEx normal samples. Normal thyroid tissue originating from GTEx was used as the target sample type for TSPG. The number of samples from each class are shown in Table 1.

**Table 1:** Samples included in the thyroid cancer gene expression matrix

Source	Sample Type	Perturbed Samples	Training Samples	Total Count
TCGA	Stage 1 THCA Tumor	10	234	244
TCGA	Stage 2 THCA Tumor	10	37	47
TCGA	Stage 3 THCA Tumor	10	85	95
TCGA	Stage 4 THCA Tumor	10	36	46
TCGA	Normal Thyroid	10	41	51
GTEx	Normal Thyroid	10	308	318

Using TSPG, we perturbed 10 samples of each type toward the target of GTEx normal thyroid tissue, including normal-to-normal perturbations to act as a baseline. This provided insight as to how THCA tumors would need to change to revert to normal thyroid tissue. TSPG outputs a matrix containing perturbation values applied to each gene in an individual sample (Table S4). A positive perturbation represents a gene that must increase its expression level to reach the average normal expression, so it is down-regulated in the tumor (tumor-downregulated). Conversely, a negative perturbation represents a tumor-upregulated gene. The terms tumor-downregulated and tumor-upregulated will be used to describe genes that were positively and negatively perturbed, respectively, by TSPG. This is meant to convey their expression level in the perturbed sample relative to the average normal thyroid expression, so even the normal-to-normal perturbations may be referred to as tumor-downregulated (positive) or tumor-upregulated (negative). During the initial training for the model used in this study, the classifier had a reported test accuracy of 0.781 and the generator had a reported perturbation accuracy of 1.000. After the training phase, there was a reported perturbation accuracy of 1.000 when the 60 randomly selected samples were perturbed.

**Fig. 1:**
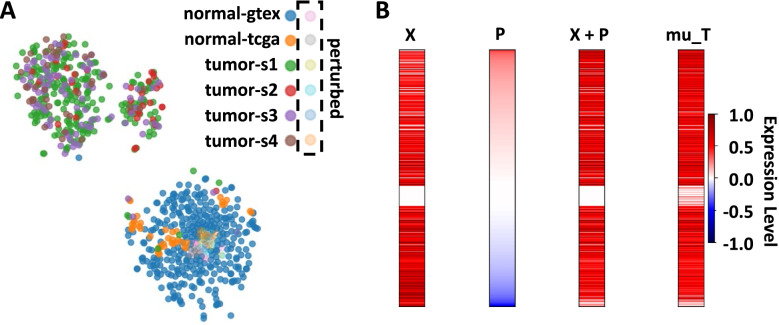
Expression profiles of 19,239 genes in THCA tumor and normal thyroid samples. **(A)** The t-SNE plot shows RNA expression profiles of normal thyroid tissue from GTEx, normal thyroid tissue from TCGA, THCA tumor samples from TCGA of various stages, and all the perturbed samples with GTEx normal thyroid tissue as the target. **(B)** The representative heatmap shows the expression vectors of one Stage 3 THCA tumor sample (X), the perturbations applied (P), the perturbed sample (X + P), and average for the target class of normal thyroid tissue from GTEx (mu_T). Missing values in the training and perturbed sample GEMs were imputed with the minimum value of the training GEM

The gene expression profiles of all THCA tumor samples and normal thyroid samples included in the study as well as the samples perturbed toward normal expression levels were visualized with a t-SNE plot (Fig. 1A). The normal thyroid tissue from both TCGA and GTEx clustered together with the perturbed samples and very few tumor samples. Most tumor samples segregated away from the normal samples. The largest difference is between the tumor and normal classes, however, there does appear to be some separation within the tumor class. The two tumor clusters appear to have similar distributions of pathologic stages.

Heatmaps were produced for each of the 60 perturbed samples. One representative heatmap for a stage 3 THCA tumor sample is shown in Fig. 1B. In order from left to right, Fig. 1B shows the expression levels of all 19,239 genes present in the GEM for the original tumor sample (X), the perturbations applied to each gene in that sample (P), the expression levels of the sample after perturbation (X + P), and the average expression levels in the target class of normal thyroid tissue from GTEx (mu_T). The red region of the perturbation box represents positive perturbations, when the expression level of a gene must be increased to reach the normal level, which indicates tumor-downregulated gene. The blue region represents negative perturbations, when the expression level of a gene must be decreased to resemble the normal level, which indicates a tumor-upregulated gene.

**Fig. 2:**
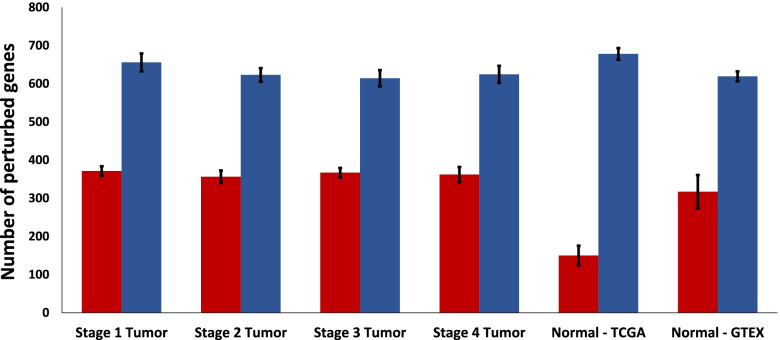
Number of genes found by TSPG to be significantly perturbed in samples within each sample type. Significantly tumor-downregulated genes, as indicated by positive perturbations toward normal thyroid tissue from GTEx, are shown in red. Significantly tumor-upregulated genes, as indicated by negative perturbations toward normal thyroid tissue from GTEx, are shown in blue. Error bars represent standard error

Figure 2 shows that, on average, there are more significantly tumor-upregulated genes than tumor-downregulated genes across all sample types. There are similar average numbers of significantly perturbed genes in both directions in all stages of THCA tumors and normal thyroid tissue from GTEx, but there were fewer significantly down-regulated genes in normal thyroid tissue from TCGA. The greatest average number of significantly up-regulated genes was identified in the TCGA normal samples.

Table 2 displays the average perturbation values for genes that were significantly tumor-downregulated or tumor-upregulated in samples of the same type. All cancer stages have similar average perturbation values of tumor-downregulated genes, while both TCGA and GTEx normal thyroid samples have lower average values. There is a less drastic difference between the tumor and normal samples for the significantly tumor-upregulated genes, but the average perturbation values are still greater in all tumors than in normal thyroid tissue.

**Table 2:** Average perturbation values of significantly perturbed genes in each sample type

Sample type	Gene set	Perturbation µ	Perturbation ϭ	Samples
**Stage 1 tumor**	Down-regulated	0.32	0.16	10
	Up-regulated	-0.26	0.19	10
**Stage 2 tumor**	Down-regulated	0.26	0.20	10
	Up-regulated	-0.19	0.20	10
**Stage 3 tumor**	Down-regulated	0.30	0.18	10
	Up-regulated	-0.23	0.19	10
**Stage 4 tumor**	Down-regulated	0.34	0.20	10
	Up-regulated	-0.27	0.20	10
**Normal thyroid (TCGA)**	Down-regulated	0.04	0.15	10
	Up-regulated	-0.18	0.19	10
**Normal thyroid (GTEx)**	Down-regulated	0.03	0.16	10
	Up-regulated	-0.12	0.18	10

**Fig. 3:**
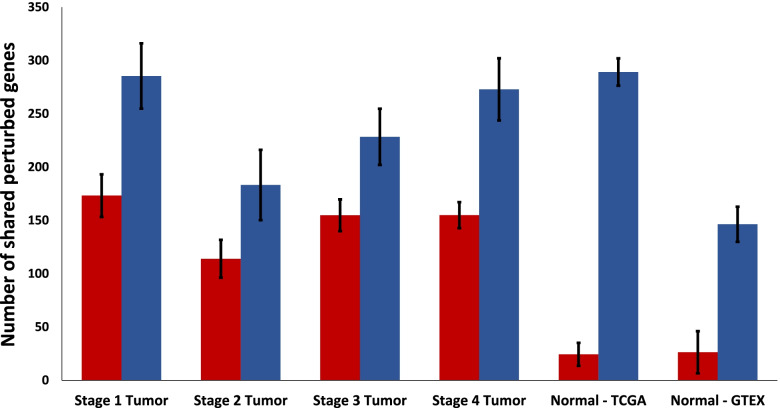
Number of shared significantly perturbed genes within each sample type. Significantly tumor-downregulated genes, as indicated by positive perturbations toward normal thyroid tissue from GTEx, are shown in red. Significantly tumor-upregulated genes, as indicated by negative perturbations toward normal thyroid tissue from GTEx, are shown in blue. Error bars represent standard error

Figure 3 displays how many genes are significantly perturbed in multiple samples from each type. The average number of tumor-upregulated genes shared between two samples of the same type is greater than that of the tumor-downregulated genes in all sample types. Normal thyroid tissue from both TCGA and GTEx have a lower average number of shared down-regulated genes than any of the tumor types. Normal thyroid tissue samples from GTEx have fewer shared up-regulated genes on average than normal thyroid samples from TCGA and all tumor types except stage 2. Stage 2 THCA tumor samples have fewer shared tumor-downregulated genes between samples than any other stage of THCA tumor samples.

**Fig. 4:**
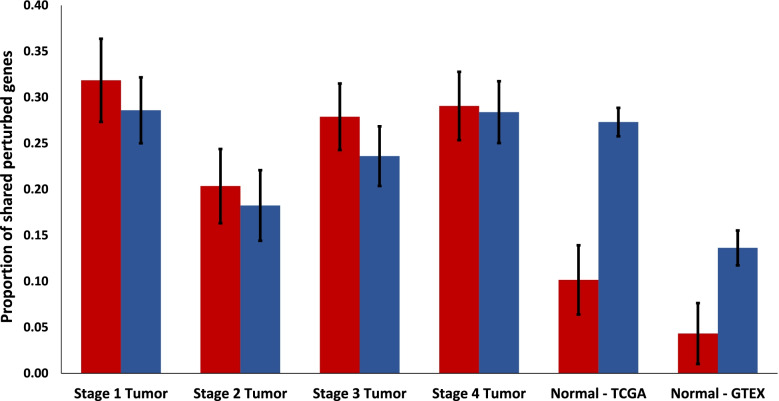
Proportion of shared significantly perturbed genes between samples, out of their unique significantly perturbed genes, within each sample type. Significantly tumor-downregulated genes, as indicated by positive perturbations toward normal thyroid tissue from GTEx, are shown in red. Significantly tumor-upregulated genes, as indicated by negative perturbations toward normal thyroid tissue from GTEx, are shown in blue. Error bars represent standard error

Figure 4 shows the average proportion of number of shared perturbed genes out of the total number of unique genes between samples of the same type. The greatest average proportion of significantly perturbed genes shared between samples within a class is about 0.32 in the tumor-downregulated genes identified in stage 1 THCA tumors. The lowest value, from down-regulated genes in normal GTEx samples, is less than 0.10. In all stages of THCA tumors, the average proportion of shared tumor-downregulated genes between two samples of the same class tend to be similar or slightly greater than that of the tumor-upregulated genes. The average proportion of shared tumor-upregulated genes is greater than that of shared tumor-downregulated genes in all normal thyroid tissue from either TCGA or GTEx, but there is a larger difference in the samples from TCGA.

The genes that were significantly tumor-upregulated or tumor-downregulated in all 10 samples of each type are seen in Table 3. There were no commonly perturbed genes in either direction among GTEx normal thyroid tissue samples, but all other sample types had at least one tumor-up-regulated and one tumor-downregulated gene common to all 10 samples. *PLA2G12B* and *RP11-73M18.2* are up-regulated in all tumor samples of any stage. All stage 1, stage 2, and stage 3 tumor samples tested had down-regulation of *SLC6A15*. Later-stage tumors (stage 3 and stage 4) showed down-regulated *REN* levels compared to normal thyroid tissue.

**Table 3:** Commonly perturbed genes in each sample type

Sample type	Tumor-upregulated genes	Tumor-downregulated genes
tumor-s1	OIT3, ARHGAP36, LRRC52, LRRK2, NT5C1A, ZCCHC16, ENTPD1, SHROOM4, CYP26C1, PLA2G12B, RP11-73M18.2, KRTAP2-3, PNPLA5, FLJ20373, SLC6A20, FIBCD1, CLPSL2, KISS1R, CST2, SERINC2	ADH4, CELA3A, GRIA1, PRM1, FAM183B, C7orf62, DEFA1, PMP2, GABRA2, MYL7, DBX2, DCD, FAM47C, CCDC168, GPR142, SLC6A15, UGT2B11, CHDC2, TSPAN19, TFF2, ADH1A, CXorf22, CLPS
tumor-s2	RP11-73M18.2, PLA2G12B	SLC6A15, PMP2, FAM47C, KCTD16, NRXN1, TNP1, ADH1A, ACSM2A, GRIA1, PPEF2
tumor-s3	AC006538.4, HAPLN1, FUT5, REN, GABRB2, PLA2G12B, RP11-73M18.2, FIBCD1, IBSP, PYDC1	VSTM2A, KIAA1239, PAPOLB, PNLIP, ADH4, CELA3A, ACSM2A, RP11-986E7.7, FAM183B, FOXD3, ADIPOQ, DEFA1, MOG, GABRA2, RP11-1220K2.2, MUSK, GPR123, AC092850.1, DCD, OTC, GPR142, TTLL6, TFF1, SLC6A15, KCNA1, HTR3C, HBCBP, ADH1A, CLPS
tumor-s4	RXFP4, SLC22A31, RP11-73M18.2, KISS1R, GRHL3, AWAT2, CSF2, GABRB2, CLPSL2, GALE, REN, HMGA2, ARHGAP36, LRP4, DUSP4, LRRK2, ADCY8, RP1-27O5.3, FIBCD1, ETV4, PLA2G2C, IBSP, CST2, SERINC2, CDH3, P4HA2, TGFA, PLA2G12B, DPP4, SLIT1, CLRN3, ENTPD1, HCN4, LIPH	C11orf74, C1orf64, PLA2R1, KIAA1239, SLC5A7, RPS6KA5, PAPOLB, PCDH11X, ZNF804B, CELA3A, GRIA1, C7orf62, ZIC2, PNLIPRP1, PMP2, MYH15, MYL7, DBX2, IPCEF1, FAM47C, GPR142, KCNA1, UGT2B11, CHDC2, TSPAN19, TFF2, TMEM174, CXorf22, FER1L6, CLPS
normal-tcga	CTD-2583A14.9, LRRIQ4, RLN1, CRYBA4, ARL14, HIST1H4B, RP11-307N16.6, C9orf135, SSX5, CLLU1, FAM19A4, RP11-571M6.15, TRIM39-RPP21, RP11-514O12.4, C9orf92, RP11-762I7.5, HIGD2B, HIST1H3A, EGR4, C1orf227, PPAN-P2RY11, AL136376.1, AL590822.2, PIH1D3, RP11-1035H13.3, CTD-2410N18.5, PIK3R2, PPY, CALR3, UPK2	PNLIP
normal-gtex	N/A	N/A

We performed functional enrichment analysis on the tumor-upregulated and tumor-downregulated gene sets for each sample type to determine the collective functions of the genes in each set. Each gene set contained genes that were significantly perturbed, in the appropriate direction, in at least one individual of the given sample type. Functional enrichment results can be seen in Table S5 and Table S6. Olfactory receptor activity and related terms were enriched in tumor-upregulated gene sets of all sample types (Table S5). There were no significant enriched terms for the tumor-downregulated gene sets from the stage 1, stage 2, and stage 3 tumor classes. Translation and related terms were enriched in the tumor-downregulated gene sets of the classes that did have significant enrichment (Table S6).

## Discussion

In this study, we used a TSPG simulation to identify tumor to normal gene expression state transitions and determine which genes exhibit aberrant expression in thyroid tumors relative to the normal thyroid gland. In essence, this is a precision medicine approach where we place an individual’s tumor (*n* = 1) into the context of other tumor samples. We previously used this approach to identify gene shifts in a single patient with Type II papillary renal cell carcinoma, but in that study we did not consider tumor stage [13]. Here, we examined 60 individuals consisting of 10 each of six different sample types, including stage 1–4 THCA tumors from TCGA and normal thyroid tissue from TCGA or GTEx. The results from the 10 samples in each class were pooled together to represent their sample type and used to investigate whether there were consistent genetic signatures for each stage of THCA. These randomly selected samples included tumors of multiple subtypes of THCA across all stages (Table S3). Future research might validate the results of this study using a limited set of samples of only one subtype of thyroid cancer to assess potential bias in pooling samples with varying primary diagnoses.

The t-SNE plot in Fig. 1A shows that the THCA tumor samples of all stages typically segregate from the normal thyroid samples. There are a few stage 1, stage 2, and stage 3 tumors that appear to cluster with the normal tissue, meaning those tumor samples have similar gene expression profiles to normal thyroid samples. These may represent tumors with very few or very small expression changes or could potentially be a sign of error in labeling or contamination with normal tissue during the RNA sequencing process. There is also evidence of two separate clusters of tumor samples containing approximately the same proportion of each stage. Further examination is needed to determine the significance and cause. Some possible causes for this discrepancy may be gender, age, and race of the individuals from whom the samples were obtained as well as the cancer subtype, or metastasis status of the samples.

The representative heatmap provided in Fig. 1B demonstrates the importance of the perturbations discussed in this study. The expression vector of the tumor sample is distinct from that of the average expression in normal thyroid tissue. However, the tumor with perturbations applied shows expression levels that are very similar to the average levels from normal thyroid samples. The t-SNE plot also supports the success of TSPG because all the samples that were perturbed with a target class of GTEx normal thyroid tissue cluster with the normal thyroid samples, indicating similar gene expression profiles (Fig. 1A).

Across all sample types, there was a greater average number of unique significantly up-regulated genes than unique significantly down-regulated genes in the original sample compared to expression in GTEx normal thyroid tissue (Fig. 2). Despite this difference, the proportion of shared perturbed genes to total unique perturbed genes between two samples of the same type was similar in the tumor-downregulated and tumor-upregulated directions across tumor samples of all stages (Fig. 4).

We expected many of the genes related to normal thyroid function would be consistently down-regulated in all tumor samples. However, this was not supported by the functional enrichment because three of the tumor classes had no significantly enriched terms for their tumor-downregulated gene sets (Table S6). Additionally, the stage 4 tumor-downregulated genes were mostly enriched for terms related to translation, none of which were unique to this gene set because they were also significant for the down-regulated genes in both normal sample types.

There were some genes that were consistently up-regulated or down-regulated in tumor samples of all or most stages, which suggests they play a role in tumorigenesis and may maintain a consistent abnormal expression level even as the tumor progresses (Table 3). Additionally, there were some unique genes found that were significantly up- or down-regulated in all tumor samples of only one stage. These are good candidate genes for future studies to investigate thyroid cancer progression to later stages.

Some genes were found to be consistently up- or down-regulated in certain sample types (Table 3). *SLC6A15* was down-regulated in all individuals with stage 1, 2, or 3 THCA. Previous studies suggest this gene acts as a tumor suppressor [25]. *PLA2G12B* was up-regulated in all tumor samples. Previous studies have observed relationships between other genes in the phospholipases A2 (PLA2) superfamily with various cancers, like over-expression of *PLA2G5* correlated with poor prognosis in patients with glioma tumors and differential expression of some PLA2 genes in normal colons and colon adenocarcinomas [26, 27]. Proteins belonging to the PLA2 superfamily are involved in metabolism, which means they may be an important indicator of normal thyroid function [28]. Therefore, disruption of PLA2 protein expression levels, as seen in all THCA tumor samples in this study, may be a potential signal of abnormal thyroid function and thyroid cancer development. *REN* was up-regulated in all later stage (stages 3 and 4) tumor samples. This gene codes for the enzyme renin, which is a component of the renin-angiotensin system [29]. Previous research indicates that the renin-angiotensin system has a role in multiple cancer types, likely due to its regulation of angiogenesis [30, 31]. Angiogenesis is an important process in cancer because increased blood flow to the tumor allows it to grow larger [32]. Since tumor size is part of the staging system, with larger tumors classified as later stages, it is possible that the up-regulation of *REN*, which induces angiogenesis, is a factor in the progression of cancer to a later stage.

There were no significantly perturbed genes that were found in all 10 GTEx normal-to-GTEx normal control samples tested (Table 3). This suggests that the perturbations applied to the GTEx normal thyroid samples, to make them appear as the target of GTEx normal thyroid tissue, were unique to individuals and represent the expected natural variations in expression among healthy individuals [33]. Whereas there were common positive and negative perturbations among the tumor samples classified as the same stage, which indicates common changes in expression that lead to the cancer phenotype. However, it should be noted that the normal thyroid samples from TCGA also had a relatively large number of common significantly up-regulated genes in all 10 samples. It is not clear whether this is a result of batch error which differentiates the TCGA and GTEx datasets, which was corrected using the methods described by Wang et al. [18]. One future experiment that may reveal more about this occurrence is to classify all normal thyroid tissue from either TCGA or GTEx together as “normal” to see if using the average expression of all normal samples as the target would eliminate the apparent difference between samples from TCGA and GTEx.

Table 2 shows that the significant perturbations applied to the normal thyroid samples, from either GTEx or TCGA, tend to be smaller than those applied to THCA tumor samples of any stage. This is a result of our method for determining the significantly perturbed genes for each sample which included those that were greater than 2 standard deviations away from the mean perturbation value within that sample. Since any of the genes in the normal samples required only small perturbations to match the average normal expression levels, Fig. 2 shows there were similar numbers of genes that were considered significantly perturbed in tumor and normal samples. There do not appear to be consistent differences in the number or value of perturbations between stages of THCA.

In this study, genes with a perturbation value of two standard deviations greater or less than the mean of all perturbations for a particular sample were deemed significantly perturbed. We were most interested in these genes that required the largest changes in expression to return to normal values because they would represent the genes that are most strongly up-regulated or down-regulated in tumors so likely have a role in tumorigenesis. However, past research has suggested that small changes in gene dosage can have a role in cancer development [34]. Future research utilizing TSPG to understand an individual’s cancer progression could consider genes with a perturbation value exceeding a threshold based on the gene’s average expression level in normal samples in order to include more subtle changes to gene expression.

Functional enrichment results for the significant tumor-downregulated and tumor-upregulated genes in each sample type revealed some similarities among the gene sets. All sample types showed enrichment for olfactory receptor activity and related terms in the tumor-upregulated gene set (Table S5). This result for the tumors aligns with previous research finding connections between abundance or stimulation of olfactory receptors and cancer [35, 36]. However, the enrichment of these functions in both normal-to-normal perturbed gene sets means these findings should be considered cautiously. We would not expect the genes perturbed in normal samples with a normal target to be enriched for relevant functions because those perturbations are expected to be random. The similar functional enrichment results for tumor-upregulated genes in all classes may indicate bias in the genes perturbed by TSPG or could potentially suggest the presence of unrecognized THCA tumor contaminating the TCGA or GTEx normal thyroid tissue. The lack of functional enrichment for down-regulated genes in tumors of stages 1, 2, and 3 was also surprising (Table S6). This is because we expected genes related to normal thyroid function to be down-regulated consistently among THCA tumor samples.

This research expands on the use of TSPG to determine how gene expression in individual tumor samples differs from that of the corresponding normal tissue. We analyzed 10 samples from each sample type perturbed toward a target of normal thyroid tissue (GTEx) to identify consistent changes in expression in different stages of THCA. This method could generate gene sets that could be used to classify tissue samples based on clinical attributes such as pathologic stage. Future experiments could explore the classification accuracy of these significantly perturbed gene sets using an existing public repository [37]. Another area to develop our understanding of differences between stages of cancer would be to look at differential expression of the identified candidate genes in samples from each stage. We also propose the use of these methods in other cancer types to determine if the differences between stages are unique to each cancer or if there are similarities.

## Conclusions

In this study, RNA expression levels from samples of THCA tumors and normal thyroid tissue were obtained from the TCGA and GTEx repositories and perturbed using TSPG with a target class of GTEx normal thyroid. These perturbations were analyzed and revealed commonly up-regulated or down-regulated genes in all or certain stages of THCA tumors. *SLC6A15* was found to be down-regulated in all stage 1–3 samples, and other studies have identified this gene as a tumor suppressor. The up-regulation of *PLA2G12B* in all samples was notable because the protein encoded by this gene belongs to the PLA2 superfamily, which is involved in metabolism, a major function of the thyroid gland. *REN* was up-regulated in all stage 3 and 4, or later stage, samples. The enzyme renin encoded by this gene, has a role in the renin-angiotensin system; this system regulates angiogenesis and may have a role in cancer development and progression. This is supported by the consistent up-regulation of *REN* only in later stage tumor samples. Functional enrichment results showed that olfactory receptor activities and similar terms were enriched for the up-regulated genes which supports previous research concluding that abundance and stimulation of olfactory receptors is linked to cancer. TSPG can be a useful tool in exploring large gene expression datasets and extracting the meaningful differences between distinct classes of data. We hope this research and future studies that stem from it will promote accurate diagnosis and appropriate treatment for THCA patients.

## Supplementary Information

**Additional file 1.** 

## Data Availability

Unified gene expression FPKM files were downloaded from https://doi.org/10.6084/m9.figshare.5330593. These processed data were originally obtained from The Cancer Genome Atlas project (https://portal.gdc.cancer.gov) and The Genotype-Tissue Expression (GTEx) project (https://gtexportal.org/home). All relevant data generated or analyzed during our study are included in this published article and its supplementary information files. If other data are required to interpret our findings, please contact the corresponding author at ffeltus@clemson.edu.

## Abbreviations

THCA: Thyroid cancer
TSPG: Transcriptome state perturbation generator
GEM: Gene expression matrix
TCGA: The Cancer Genome Atlas
GTEx: Genotype-Tissue Expression
AJCC: American Joint Committee on Cancer
PLA2: Phospholipases A2
MAPK: Mitogen-activated protein kinase
MSigDB: Molecular Signatures Database
TSV: Tab-separated value
GO: Gene Ontology
IPR: Interpro
KEGG: Kyoto Encyclopedia of Genes and Genomes
